# ClinSeK: a targeted variant characterization framework for clinical sequencing

**DOI:** 10.1186/s13073-015-0155-1

**Published:** 2015-03-31

**Authors:** Wanding Zhou, Hao Zhao, Zechen Chong, Routbort J Mark, Agda K Eterovic, Funda Meric-Bernstam, Ken Chen

**Affiliations:** Department of Bioinformatics and Computational Biology, the University of Texas MD Anderson Cancer Center, Houston, TX 77030 USA; Department of Hematopathology, the University of Texas MD Anderson Cancer Center, Houston, TX 77030 USA; Department of Systems Biology, the University of Texas MD Anderson Cancer Center, Houston, TX 77030 USA; Institute of Personalized Cancer Therapy, the University of Texas MD Anderson Cancer Center, Houston, TX 77030 USA; Department of Investigational Cancer Therapy, the University of Texas MD Anderson Cancer Center, Houston, TX 77030 USA

## Abstract

Applying genomics to patient care demands sensitive, unambiguous and rapid characterization of a known set of clinically relevant variants in patients’ samples, an objective substantially different from the standard discovery process, in which every base in every sequenced read must be examined. Further, the approach must be sufficiently robust as to be able to detect multiple and potentially rare variants from heterogeneous samples. To meet this critical objective, we developed a novel variant characterization framework, ClinSeK, which performs targeted analysis of relevant reads from high-throughput sequencing data. ClinSeK is designed for efficient targeted short read alignment and is capable of characterizing a wide spectrum of genetic variants from single nucleotide variation to large-scale genomic rearrangement breakpoints. Applying ClinSeK to over a thousand cancer patients demonstrated substantively better performance, in terms of accuracy, runtime and disk storage, for clinical applications than existing variant discovery tools. ClinSeK is freely available for academic use at http://bioinformatics.mdanderson.org/main/clinsek.

**Electronic supplementary material:**

The online version of this article (doi:10.1186/s13073-015-0155-1) contains supplementary material, which is available to authorized users.

## Background

A major objective of clinical genomics is to translate the knowledge and technologies that are established in a discovery setting, for example, large-scale cancer genome sequencing, into a clinical setting to benefit individual patients [1]. Despite the tremendous progress in discovering mutations in patients, only a small set of variants have been associated with causal clinical evidence and therefore have been regarded as actionable in clinics [2]. For example, the standard panel for screening cystic fibrosis as recommended by the American College of Medical Genetics is composed of only 23 mutations in cystic fibrosis transmembrane conductance regulators [3]. Even after accounting for all the mutations reported for the disease up to 2014, the number of mutations is still under 2,000 [4]. In another example, three mutations in HEXA account for over 92% of affected Tay-Sachs patients [5]. The stark contrast between the mutations present and the mutations that physicians could respond to motivates a re-structure of the bioinformatics workflow that concentrates variants that lead to known clinical consequences.

The current paradigm for clinical variant characterization based on next generation sequencing was designed for discovering new variants [6] unknown to the scientific community. It involves aligning every read to the human reference assembly, discovering mutations at every position in the reference, and providing functional annotations through existing algorithms [7]. Tools developed under such a paradigm not only suffer from the ‘big-data challenge’ [8], which could hinder application in hospital settings that lack powerful computing infrastructure, but also are likely to report many variants of unknown clinical significance. In addition, they may produce suboptimal results at sites that harbor actionable mutations, partially because of the criteria implemented for controlling global false positives. The increasing use of next generation sequencing for genomic testing [9] warrants the development of a new set of tools that operate under a paradigm that emphasizes characterization on important clinical targets.

To answer the demand, we have designed and implemented ClinSeK, a bioinformatics tool that focuses computational power on clinically relevant sites while avoiding investigating mutations that are non-actionable, hence ameliorating the big-data challenge. The tool adapts the entire arsenal of variant characterization techniques used in a variety of applications to the targeted paradigm. Compared with existing tools designed for each separate application, ClinSeK achieves tremendous reduction in computational cost with higher sensitivity and comparable accuracy in the target zone. ClinSeK provides software-level target capture to supplement existing sequencing-level techniques [10].

## Methods

**Figure 1:**
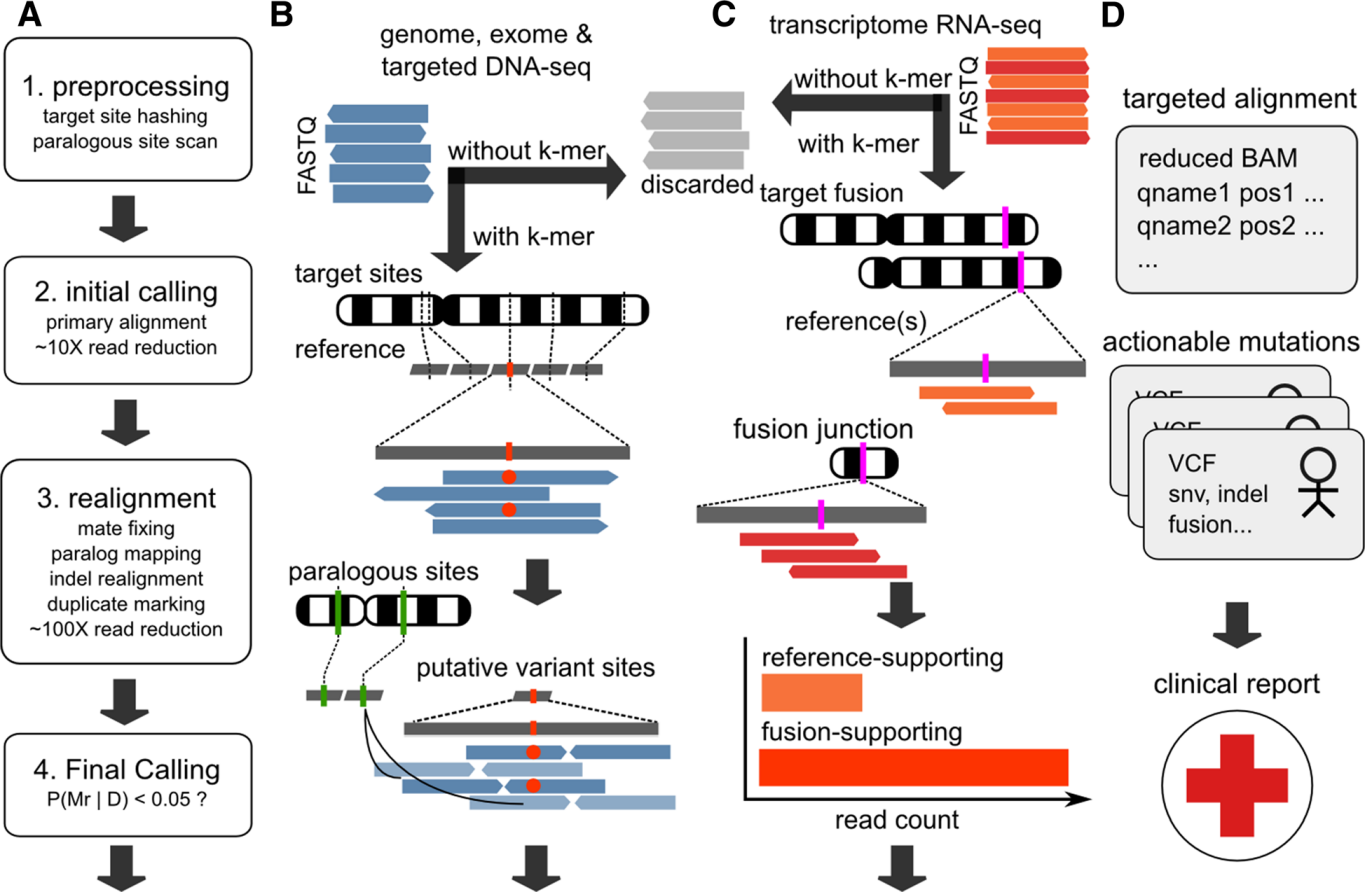
Schematic overview of ClinSeK. **(A)** The four major steps of the ClinSeK workflow for analyzing single nucleotide variants (SNVs) and insertions and deletions (indels) from DNA-sequencing data. **(B)** Illustration of k-mer screening, targeted alignment and variant calling. Sequencing reads (blue arrows) in raw FASTQ files are screened for presence of k-mers created from target sites of interest (dark, vertical dashed lines), which are predefined based on variant databases such as ClinVar and COSMIC. Those that do not contain any target k-mers (grey arrows) are discarded. Those associated with a target site (red vertical bar) are aligned against corresponding local reference sequences (grey horizontal bars) with potential variants (red dots) identified. Reads were realigned with mates (arrow in opposite directions) and against paralogous sites (green vertical bars) from other chromosomes. Variants are finally called from reads of high mapping quality (dark blue arrows). **(C)** Illustration of ClinSeK targeted breakpoint analysis. DNA or RNA sequencing reads are screened for presence of k-mers in the reference and in the variant alleles near the breakpoints or fusion junctions. Those that do not contain any target k-mers are discarded. The remaining ones are preferentially aligned to the wild-type reference (orange arrows) and to the fusion breakpoint (magenta bar) sequence (red arrows) and are counted and compared. **(D)** ClinSeK output. Reads and their alignments at the target sites are output in BAM files. Variants are output in VCF format and are further included in the clinical report.

Starting from the short reads sequenced from a patient sample and a list of clinically relevant variant sites, ClinSeK aligns and analyzes only the reads that are relevant to the given target sites (Figure 1A). This fundamentally differentiates ClinSeK from base-to-base discovery pipelines composed of aligners such as BWA [11] and downstream variant callers such as GATK [12] and MuTect [13]. The computational cost of ClinSeK depends on the number of potential clinical targets to be assessed. The total number of mutations that are likely to be associated with all the known clinical phenotypes in ClinVar [14] is on the order of 100,000 (79,355 as accessed on 30 April 2014). Categorized by pathological conditions, many rare yet well-characterized genetic disorders are associated with a handful of mutations [3,5]. For example, 18 mutations in ClinVar are related to sickle-cell anemia [14]. Ten mutations are found related to familial dysautonomia [14]. Complex common diseases such as diabetes and cancer include more causal mutations. But even for cancer treatment assignment, only several hundred somatic mutations are currently viewed as actionable [15,16]. By analyzing only reads relevant to the sites that harbor these mutations (single nucleotides for single nucleotide substitutions and insertions, and genomic regions for deletion and multiple nucleotide substitutions), one can potentially achieve a substantial reduction in computational cost.

A naïve approach that directly aligns the reads to a ‘squashed’ reference that contains only target sites would lead to many false alignments and overestimation of alignment quality. Therefore, the key challenge is to ensure the set of relevant reads obtains both globally and locally optimal alignments without referencing the entire reference assembly. ClinSeK accomplishes this goal in the following steps (Figure 1A,B). First, ClinSeK divides the reference sequences over the target sites into a k-mer (a nucleotide sequence of length k) library and creates a catalogue of paralogous sites that are homologous to the target sites in the reference genome. The set of target sites can be easily updated to accommodate new variants of interest. The size of the k-mer is chosen to achieve a good balance between alignment sensitivity and efficiency. Second, ClinSeK identifies ‘target reads’ that contain at least one k-mer in the library and discarded reads that do not contain any k-mer in the library. It obtains an initial Smith-Waterman alignment of the target reads to the target site. It then identifies sites that are spanned by a minimal amount of reads that support variant alleles. This narrows the scope of analysis to the subset of target sites that likely contain variants. Optionally, ClinSeK can output the variant status at all target sites, allowing users to distinguish true negative sites from those lacking coverage. Third, ClinSeK realigns reads at putative variant sites by including their mates and factoring in their multi-alignments to pre-identified paralogous sites (Figure 1A,B). In addition, ClinSeK scans for insertions and deletions (indels) around the target sites and performs a local dynamic programming alignment if an indel haplotype can be reconstructed. ClinSeK also implements a refined duplicate read marking algorithm that is aware of not only alignment positions but also base identities and qualities (Additional file 1). Finally, a Bayesian approach is applied to estimate the probability of variants given the aligned reads. To ensure accuracy, only reads with high mapping qualities (>30) contribute to the final variant calling. In contrast to conventional analysis pipelines, ClinSeK tightly integrates alignment and variant calling, which effectively reduces computational cost while improving the quality of the data at the sites of interest.

Similar to targeting single nucleotide variations (SNVs) and indels, ClinSeK can target genomic structural variation from DNA-seq data or gene fusion breakpoints from RNA-seq data when breakpoint sequences are provided (Figure 1C). ClinSeK contrasts reads that are preferentially (as judged by alignment score) aligned to alternative alleles that contain breakpoints to ones preferentially aligned to wild-type references, similar to methods that quantify differential expression across different isoforms or genes [17]. The breakpoint sequences spanning pathogenic fusion junctions are usually available [18] or can be derived from genome or transcriptome assembly [19,20].

ClinSeK takes as input FASTQ files. For SNVs and indels, ClinSeK outputs a reduced BAM file which contains the alignment of reads to the target sites and a VCF file which contains the list of variants at the target sites and their characteristics (Figure 1D). These files are orders of magnitude smaller than those produced by the standard discovery pipeline and more conveniently applied to clinical decision-making.

### Targeted alignment and handling of paralogous sites

The specificity of ClinSeK short read alignment highly depends on the balance of its alignment sensitivity against the sensitivity to paralogous sites. At extremes, the entire genome should be included as potential paralogous sites. This is the approach taken by many popular global aligners based on full-text indexing [21,22]. Optimized for speed of aligning reads to the entire genome, such full-text indexing is unnecessarily demanding in memory usage and requires online reconstruction of the suffix array, when compressed, for targeted alignment. Instead, we adopted the traditional hashing-based method, which is similar to MAQ [23]. The availability of any prior knowledge of paralogous sites is necessary for ClinSeK alignment so that false alignments can be discerned. Hence, before it can be applied to sequencing data, ClinSeK scans the reference genome to obtain information of paralogous sites that share sequence similarity with the target sites. This scanning needs to be done only once on each compilation of target sites and can be reused upon processing different samples.

A keen recognition of paralogous sites is crucial for ClinSeK to prevent false alignment while maintaining high sensitivity in read alignment. As illustrated in Additional file 2, false alignments are nonexistent when both alignment sensitivity and paralogy sensitivity are low or when both are high (Figure S1A,C in Additional file 2). False alignment (green triangle) emerges when paralogy sensitivity is not high enough (even if higher than alignment sensitivity) (Figure S1B in Additional file 2). In ClinSeK, a site is considered paralogous (which is defined purely on the basis of sequence similarity) if one of the three 48-bp stretches can be aligned to the target site with fewer than 4 mismatches or with a mix of indels with identical alignment scores (Figure S1D in Additional file 2). Figure S1E in Additional file 2 shows the number of paralogous sites identified for three different target site groups studied in this paper: 1) the AmpliSeq64 mutation sites; 2) ClinVar mutation sites restricted to 202 cancer genes; and 3) the whole set of ClinVar variant sites. For ClinSeK, segments with too many paralogous sites (>50 by default) are excluded from analysis. They are considered to be of low complexity and too risky for clinical use. Most sites from ClinVar (>95% and >99.9% if restricted to 202 cancer genes) contain fewer than 50 paralogous sites and are amenable to ClinSeK processing.

**Figure 2:**
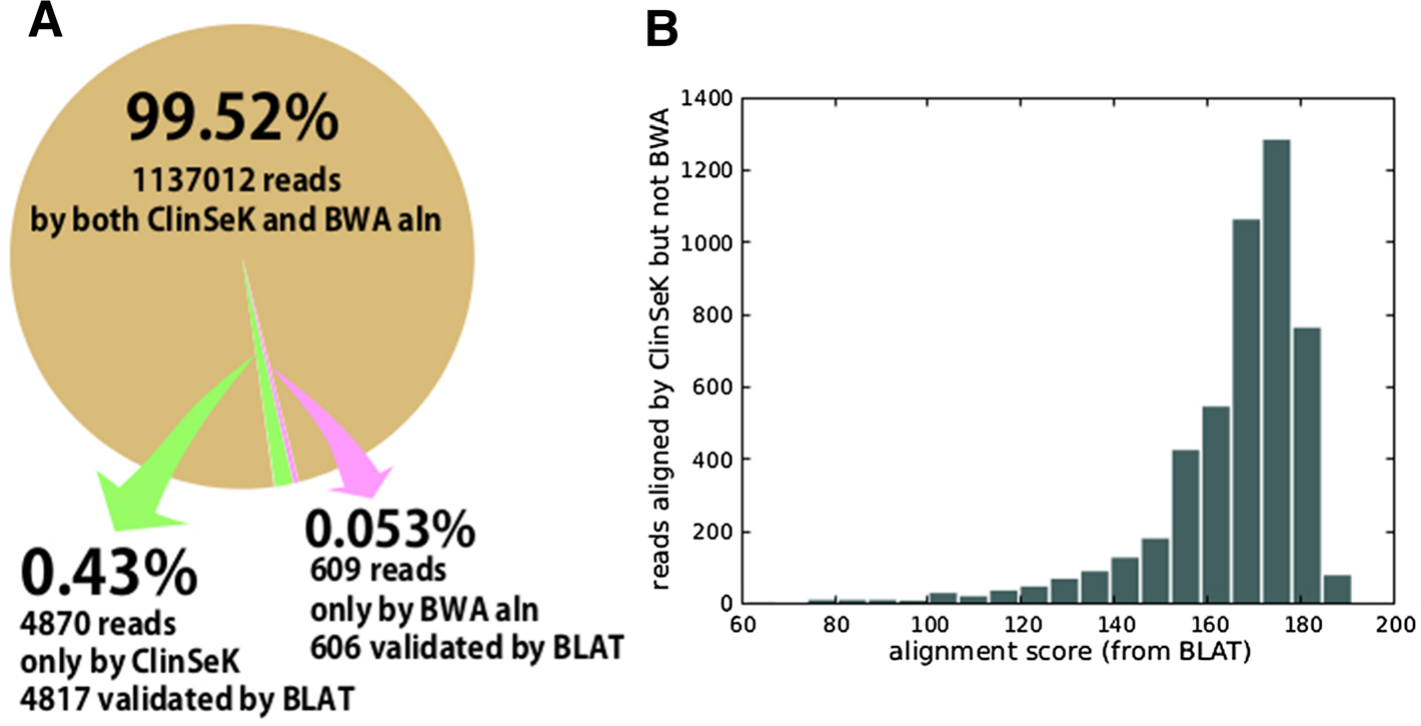
Comparison of ClinSeK alignment with BWA. **(A)** Comparison between ClinSeK and BWA alignment. The numbers are reported from 1,000 sites randomly chosen from the ClinVar database and from 700 samples. Brown color indicates the overlap between ClinSeK and BWA alignments. Green color indicates reads aligned to the target site by only ClinSeK; pink color indicates reads aligned by only BWA. **(B)** Alignment score distribution of read alignment by only ClinSeK but not BWA aln. The alignment score is calculated by BLAT, with the maximum score of 200 for reads as long as 100 bp.

The primary read alignment is done by seed-anchoring and local alignment. To guarantee high sensitivity of reads to the reference, multiple seeds are selected around the target sites. By default, for each target site, ClinSeK hashes twelve 25-mer reads (seeds) evenly positioned such that at least two mismatches are tolerated in any reads that cover the target site given a read length longer than or equal to 100 bp. Upon each occurrence of a seed sequence in a read, the read is compared with the local reference sequence around the seed. To balance speed and optimality, this is done in two steps. First, seeds are extended in two directions to seek a complete read match tolerating at most one mismatch. Upon any failure, local dynamic programming is then performed to obtain the optimal alignment under a given scoring scheme. Alignments with too many sequence dissimilarities on high-quality bases are discarded. As is shown in Figure 2, this combination of seeding strategy and local alignment makes ClinSeK alignment highly accurate (Figure 2A) and sensitive even to low quality reads (Figure 2B) compared with BWA aln (see Additional file 1 for details of the comparison). Such increased sensitivity in alignment helps alleviate potential bias in the reference sequence, particularly when analyzing samples that come from different ethnic background.

ClinSeK is designed to operate on paired-end reads of lengths ranging from 75 bp to 500 bp, which is currently the most widely used platform for clinical sequencing. Traversing raw FASTQ reads, ClinSeK records the number of reads that indicate genetic variation at each target site. Only sites containing over a minimum number of variant reads (default of three) are considered in the subsequent analysis (initial variant calling). Because genetic variations are rare, this practice greatly reduces 1) the number of sites where mate-reads need to be aligned; 2) the number of inserts that need to be further analyzed; and 3) the number of reads whose alignments need to be stored, without losing information of potential variant reads. Note that, after this stage, the remaining sites can still be non-variant due to wrongly mapped reads from paralogous sites. Given the variant status known from the initial variant calling, the second traversal of the reads effectively skips most reads and aligns only mate-reads around the putative variant sites if their alignments have not been completed. With the alignment of the full insert, further comparison with the pre-identified paralogous sites can be achieved. We assigned a mapping quality to every alignment of each insert to quantify the strength of the evidence that the insert could provide for the presence of the corresponding allele. Following the work of Li and Durbin [23], the mapping quality is defined as the Phred-scaled probability that the insert was sequenced from a different genomic location. In practice, it is computed using *Q*_*i*_ = -10 log_10_1 - 10^-*ms*(*i*)^/∑_*i*_10^-*ms*(*i*)^, with *i* looping over all the alignments of the insert of sufficiently high alignment score (with a default threshold of the 90th percentile of alignment scores computed for each read from bases of quality over 20). The term *ms*(*i*) denotes the sum of the base qualities of all the mismatched bases in alignment *i*. Based on this formula, reads mapping to sites that have multiple paralogous sites will have very small (near 0) mapping quality. This design effectively limits the false positive rates for sites in repetitive regions.

### Targeted breakpoint analysis

The breakpoint analysis is carried out by first hashing for each structural variation breakpoint, the alternative breakpoint sequence assembly and the corresponding reference sequence(s) around the breakpoint (Additional file 3). Every read, upon anchoring through a seed sequence, is aligned to both the alternative sequence and its corresponding reference sequences(s). For each structural variant breakpoint, we keep a record of the number of reads preferentially aligned to the reference allele and those preferentially aligned to the alternative allele. We conclude a breakpoint if there are a large number of reads that support the alternative allele. Our methods apply to both DNA-seq or RNA-seq data dependent on whether the reference allele is constructed from the genome or a transcriptome.

### Indel realignment

Penalizing gaps in the alignment scoring scheme may cause collective bias in SNP calling close to a *bona fide* indel [12,24]. To mitigate false positive SNV calling around indels and also to improve the measurement of the allele frequency of these indels, ClinSeK enumerates all the indels from the local read alignment. Alternative gapped haplotypes are then reconstructed from well-supported indels. ClinSeK samples from the set of all indel events subsets of non-overlapping but adjacent (within twice the read length) indel events and enumerates (exhaustively) all putative haplotypes resulting from at most m events (with m default of two). Every read around the target site is realigned against the putative alternative gapped haplotypes. If the resulting alignment score is higher than the alignment score against the ungapped haplotype, a new alignment is then reconstructed by composing the alignment of the read to the gapped haplotype with the mapping between the gapped haplotype and the reference genome. The reconstruction combines contiguous insertions, deletions and substitutions and produces a valid CIGAR string. Currently, ClinSeK only realigns indels that are found in the initial alignment. Additional files 4 and 5 show two examples of the correction of false positive somatic mutations using this approach.

### Variant calling and genotyping

ClinSeK identifies the variant status through a Bayesian model parameterized by 1) contamination (default to 0.01); 2) sequencing error rate (default to 0.001); and 3) empirical mutation rate (default to 0.001, the average of genetic diversity in normal human population [25]). The contribution of these default priors to the final scores is minor and can be adjusted through command line for particular use-cases. To characterize the variant status, we consider two classes of models: 1) reference model *M*_*r*_, where all variants are explained by sequencing error or contamination; and 2) variant model *M*_*v*_, where variants are explained jointly by sequencing error and the presence of a variant allele at fraction *f*. The *P*-value of calling a variant is computed by *P*(*M*_*r*_|*D*) = *P*(*D*|*M*_*r*_)*P*(*M*_*r*_)/*P*(*D*). See Additional file 1 for details on computing the value.

For germline mutations, likelihoods of the three genotypes are also computed. Let *g* denote the genotype of the mutation, that is, $=\left\{0,\frac{1}{2},1\right\}$, representing the homozygous reference, the heterozygous variant and the homozygous variant, respectively. The genotype is called by maximizing the following posterior probability accounting for sample contamination. *D*_*N*_ denotes the read counts for each allele in the normal sample:

$$ ar\mathit{\mathsf{g}}ma{x}_{g\in \left\{0,1/2,1\right\}}P\left(\mathit{\mathsf{g}}\left|{D}_N\right.\right)=ar\mathit{\mathsf{g}}ma{x}_{g\in \left\{0,1/2,1\right\}}C\mathit{\mathsf{g}}{\displaystyle {\int}_{\max \left(\mathit{\mathsf{g}}-{C}_{max},0\right)}^{\min \left(\mathit{\mathsf{g}}+{C}_{max},1\right)}P\left({D}_N\left|\mathit{\mathsf{g}},c\right.\right)dP(c)}, $$

where $C\mathit{\mathsf{g}}=1/\left( \min \left(\mathit{\mathsf{g}}+{C}_{max},1\right)- \max \left(\mathit{\mathsf{g}}-{C}_{max},0\right)\right)$. *c* is the dummy variable for integrating over all possible values of sample contamination. The probability of sample contamination is assumed to be a uniform probability from 0 to *C*_*max*_. See Section 2 of Additional file 1 for details in calculating $P\left({D}_N\left|\mathit{\mathsf{g}},\mathit{\mathsf{c}}\right.\right)$.

Since we are genotyping patient samples, which are more likely to contain relatively recurrent mutations on the target site, the probability of observing heterozygous and homozygous variant sites is higher than that expected from a random site in a normal population. A uniform prior for the three genotypes is chosen as default.

### Somatic calling using matched tumor and normal samples

Four models are considered in explaining the read counts in both tumor and matched normal samples. Let symbol *M*_*ij*_ denote the model where *i*, *j* ∈ {*v*, *r*} labels whether the variant or only the reference allele is present in the normal and tumor samples, respectively. Model *M*_*rv*_ suggests a somatic mutation under the common definition; that is, variants exist in tumor samples but not normal samples. The other three models, *M*_*rr*_, *M*_*vv*_ and *M*_*vr*_, respectively, represent cases where 1) there are no variants in both sample; 2) a germline mutation is present (in both samples); and 3) a form of loss of heterozygosity takes place, in which case a germline variant is lost in the tumor sample.

The posterior probability of somatic mutation is hence given by:

$$ \begin{array}{c}P\left({M}_{rv}\left|D\right.\right)=\frac{P\left(D\left|{M}_{rv}\right.\right)P\left({M}_{rv}\right)}{{\displaystyle {\sum}_{i,j}P\left(D\left|{M}_{ij}\right.\right)P\left({M}_{ij}\right)}}\\ {}=\frac{P\left({D}_n\left|{M}_{rv}\right.\right)P\left({D}_t\left|{M}_{rv}\right.\right)P\left({M}_{rv}\right)}{{\displaystyle {\sum}_{i,j}P\left({D}_n\left|{M}_{ij}\right.\right)}P\left({D}_t\left|{M}_{ij}\right.\right)P\left({M}_{ij}\right)}\end{array} $$

Calculation of the likelihood follows the procedure used in variant calling. The somatic mutation score reported by ClinSeK is the Phred-scaled *P*(*M*_*rv*_|*D*).

### Base-to-base discovery pipeline

To evaluate ClinSeK in comparison with standard approaches to variant characterization, we set up a base-to-base sequence analysis pipeline composed of alignment, variant calling and other processing such as duplicate marking, indel realignment and filtering. We completed the alignments using BWA [26] and marked duplicate reads using Picard [27]. We called single nucleotide variation using either VarScan2 [28] (version 2.3.2) or GATK [12] (version 3.1.1). For matched tumor/normal samples, VarScan2 labels somatic mutations that are used in the comparison with ClinSeK for somatic variant calling. We also used MuTect [13] (version 1.1.4) to detect somatic mutations. For MuTect, only mutations labeled ‘KEEP’ were considered in our comparison.

Our research was approved by the MD Anderson Cancer Center Institutional Review Board under protocol #PA11-0852. Exon-sequencing data for testing ClinSeK can be downloaded from the Short Reads Archive [SRA: SRP033243]. ClinSeK was compared with other tools using the targeted exome sequencing data of 1,049 pairs of tumor and matched normal samples [29]. For detailed instructions and for downloading a set of precompiled target sites containing actionable or putative driver mutations in cancer, please access our website at [30].

## Results and discussion

We assessed ClinSeK using the targeted exome sequencing data from 1,049 pairs of tumor and matched normal samples [29]. A set of 565 somatic mutations in this data set had been previously independently ascertained using a CLIA-compliant amplicon-based hotspot sequencing platform (Ion AmpliSeq64 produced by Life Tech, Grand Island, NY 14072, USA). This provided a ‘reference standard’ for comparing the sensitivity of ClinSeK against those of other tools in an unbiased manner.

**Figure 3:**
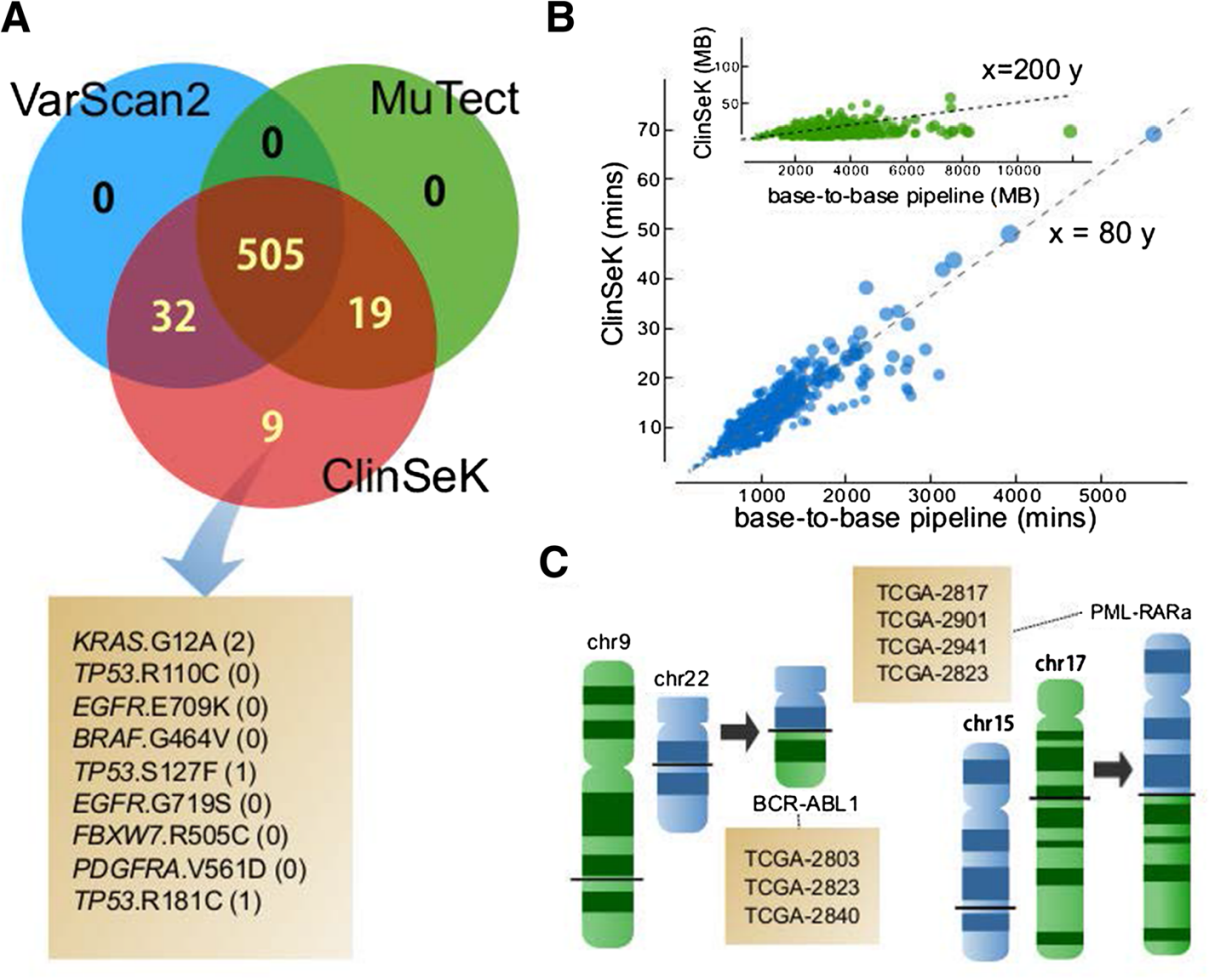
ClinSeK performance in analyzing DNA-seq and RNA-seq data. **(A)** Comparison of ClinSeK, VarScan2 and MuTect sensitivity in characterizing somatic mutations from 1,024 targeted exome-sequenced tumor and normal pairs. Text box lists CLIA-validated somatic mutations detected only by ClinSeK. **(B)** Comparison of ClinSeK and the base-to-base pipeline in runtime (blue dots) and data storage (green dots). Dashed lines correspond to 80× and 200× respective reductions in runtime and storage. Dot sizes are proportional to the number of reads sequenced from each sample. **(C)** Illustration of ClinSeK gene fusion detection results on The Cancer Genome Atlas (TCGA) samples. Black horizontal bars indicate breakpoints. Text boxes list TCGA sample names, in which the corresponding fusion is detected.

We targeted ClinSeK to the set of 719 variant sites on the AmpliSeq64 panel and identified 1,006 somatic mutations under the default setting. For comparison, we aligned the same set of reads to the human genome assembly GRCh37 using BWA-aln and called somatic mutations using VarScan2 [28] and MuTect [13] under default parameters. ClinSeK successfully detected all of the 565 known somatic mutations (100% sensitivity), while MuTect and VarScan2 detected 524 (92.7%) and 534 (94.5%), respectively (Figure 3A). The nine mutations missed by both VarScan2 and MuTect included potentially important mutations such as *KRAS* G12A, *EGFR* E706K and *TP53* R181C (Additional file 6). Six of the missed calls are the only mutations identified in the corresponding tumor samples and are thus potentially critical to clinical decision-making. Inspection of read counts for alternative alleles in the matched normal samples revealed that MuTect missed these mutations likely due to its high expectation on the purity of normal samples (Additional file 7). A recent study indicated that blood DNAs of cancer patients could contain somatic mutations [31]. On the other hand, investigation of local mutation context reveals that Varscan2 missed them due mainly to *ad hoc* filtering of mutations near other variants such as co-segregating SNPs, multi-nucleotide variants or indels (Additional file 8). We also found that improvement in ClinSeK alignment contributed to increased sensitivity (Additional file 1). Notably, ClinSeK took only 10 to 30 minutes to analyze each sample (with a median of 25 million reads), while the standard pipelines took over 1,000 minutes and in some cases over 2,000 minutes (Figure 3B). The resulting files (BAM and VCF) output by ClinSeK are substantially smaller than those obtained from standard pipelines. Such a significant reduction in runtime (80-fold) and data storage (200-fold) makes ClinSeK uniquely suitable for clinical applications (see Additional file 1 for detailed comparison of runtimes using different sites as target and data from other sources). The variant allele fraction calculations are highly concordant with those calculated by VarScan2 and MuTect (Additional files 9 and 10).

To assess the specificity of variant callers, we conducted independent sequencing experiments on the same sample (technical replica) from each of 16 normal tissue samples. We targeted 719 clinically actionable variant sites on the AmpliSeq64 panel and applied ClinSeK and MuTect to each pair of technical replicas by treating one technical replicon as a tumor and the other as the matched normal tissue. Any somatic mutations identified from this set would be false positives, as explained in a previous study [13]. We found no false positive variant call (100% specificity).

ClinSeK can be applied to detect either somatic or germline variants, depending on the configuration. A comparison of ClinSeK with GATK on variant calling from the normal samples showed comparable accuracy (approximately 99.6% concordance rate) of germline variant calling between the two tools (Additional file 1). On this data set, ClinSeK achieves higher sensitivity with high specificity compared with MuTect for somatic mutation detection and GATK for germline mutation calling.

In addition to SNVs and indels, we also validated the ability of ClinSeK to identify somatic structural variation breakpoints. We applied ClinSeK to test the presence of pathogenic *BCR-ABL1* or *PML-RARa* fusions in the RNA-seq data of six samples of acute myeloid leukemia from The Cancer Genome Atlas [32] (dbGAP: phs000178.v7.p6). The fusion breakpoint sequences were previously available in an mRNA breakpoint library obtained through transcriptome assembly [33]. ClinSeK was able to successfully identify all seven previously known gene fusions in all the six samples within a timeframe of 10 minutes per sample (Figure 3C).

ClinSeK identifies variants only at the targeted sites and does not discover any novel variant. For discovery or prospective studies, ClinSeK can be used in conjunction with other base-by-base tools to increase the detection sensitivity of clinically important variants. As novel clinically important variants are being discovered, users can easily update ClinSeK libraries to include them. Establishing the clinical utility of novel variants involves lengthy and costly clinical trials, which usually take years, while clinical decision-making demands rapid turnaround in days or minutes. The development of ClinSeK separates these two distinct tasks and accelerates the translation of robust clinical genomic knowledge to today’s patients.

## Conclusions

The development of ClinSeK offers a software-level solution to the ever-increasing demand for efficient and accurate variant characterization in clinical sequencing. It is software designed starting from a set of clinically actionable sites and comprehensively interrogating these sites efficiently without investing computational resource to sites that are of no clear clinical relevance. It is dedicated to characterizing variants in clinical settings where only a limited set of relevant mutations needs to be quickly characterized with the highest possible accuracy. It allows clinical variant characterization be achieved much faster (minutes compared to hours, and hence ameliorating the big-data challenge) and to a higher accuracy than a base-to-base discovery pipeline conducted in current clinical sequencing applications.

ClinSeK can be applied to detecting the majority of variants, including SNVs, indels, structural variants and gene fusions from whole genome, whole exome, targeted exome and transcriptome sequencing data. ClinSeK is available for academic use at [30].

## Additional files

Additional file 1:
**Supplementary notes and methods.** 1) Comparison of ClinSeK targeted alignment with BWA aln. 2) Computing ClinSeK variant score. 3) Read pileup and calculation of allele support. 4) Duplicate insert marking. 5) Somatic mutation detection by ClinSeK. 6) Variant calling on >1,000 normal samples. 7) Implementation and memory footprint. 8) Run time on targeted exome sequence samples. Additional file 2: Figure S1. Schematic illustration of paralogous scanning. Distance between symbols (triangles and stars) reflects the edit distance between sequences. Red star, target site sequence; red triangle, read sequenced from target site; green star, paralogous site sequence; green triangle, read sequenced from paralogous site. Size of the dashed circle indicates the sensitivity in identifying sites paralogous to the target site. Size of the solid circle indicates the sensitivity in read mapping. Greater circle size represents higher sensitivity. **(A)** Low sensitivity in read mapping and low sensitivity in paralogous scanning. No false positive exists (no green triangle in the solid circle), but there is a missing read alignment (red triangle outside the solid circle). **(B)** High sensitivity in read mapping and low sensitivity in paralogous scanning. False positives occur (green triangle in a solid circle). **(C)** High sensitivity in read mapping and high sensitivity in paralogous scanning. Neither false positives nor false negatives occur. **(D)** Definition of paralogous sites. Blue horizontal bar: sequence stretch on which fewer than four mismatches exist. Green horizontal bar: target sequence (top) and paralogous site sequence (bottom). We use a default scoring system with affine gap penalties of 2:3:1 for [mismatch]:[gap opening]:[gap extension]. **(E)** The number of target sites amenable to ClinSeK processing is shown in blue and the number of paralogous sites identified is shown in pink. Three different target site sets are studied: 1) AmpliSeq64; 2) ClinVar sites restricted to 202 cancer genes; 3) ClinVar sites. Additional file 3: Figure S2. Schematic diagram of fusion detection. The number of reads aligned to alternative fusion breakpoint assembly is contrasted with the number of reads aligned to the reference sequence around the breakpoint. Green and blue indicate the two reference sequences involved in the gene fusion. Narrower bars stand for short reads. The color of the reads indicates sequence similarity with the reference sequence. Additional file 4: Figure S3. Elimination of false positive SNVs by indel realignment. Sample: IPCT-CH-4335-Tumor-945; site: chr5:112175216. **(a)** Before indel realignment, a false positive mutation (T) was present. **(b)** After indel realignment, the false positive is eliminated. Additional file 5: Figure S4. Elimination of false positive SNVs by indel realignment Sample: IPCT-CH-4522-Tumor-1082; site: chr5:112175423. **(a)** Before indel realignment, a false positive mutation (T) was present. **(b)** After indel realignment, the false positive is eliminated. Additional file 6: Table S1. List of validated mutations detected by only ClinSeK and missed by VarScan2 and MuTect, together with potential causes of missed mutations as reported from MuTect in rejecting these somatic mutations. Additional file 7: Table S2. List of validated mutations missed by MuTect but reported by ClinSeK and VarScan2. Potential causes of missed mutations are listed in column 6. It can be seen from these tables that MuTect misses high frequency somatic mutations because of a low-level alternative read count in the normal sample. Additional file 8: Table S3. List of validated mutations missed by Varscan2 but reported by ClinSeK and MuTect. Potential causes of missed mutations obtained from manual inspection are listed in column 5. VarScan’s false negatives are primarily due to either 1) mutations that are found in regions where there are other mutations nearby (where mutations are locally clustered); or 2) the allele frequency being below a certain cutoff. Abbreviations: DNV, di-nucleotide variation; SNV, single nucleotide variation; TNV, tri-nucleotide variation. Additional file 9: Table S4. Comparison of variant allele fraction (VAF) calculation on 719 actionable mutation sites from 1,049 paired tumor deep sequencing data sets. Sample names are omitted. Additional file 10: Figure S5. Comparison of variant allele frequencies. **(A)** The variant allele frequencies are estimated by VarScan2 (x-axis) and ClinSeK (y-axis) from 3,472 genetic variants in 46 deep sequenced normal samples. **(B)** Variant allele frequencies estimated by GATK (x-axis) and ClinSeK (y-axis) from 3,467 germline mutations in 46 deep sequenced normal samples.

## Abbreviations

bp: base pair
SNV: single nucleotide variation
