# Supplementary material for: ClinSeK: a targeted variant characterization framework for clinical sequencing
Source: Genome Med. 2015 Mar 31;7(1):34. doi: 10.1186/s13073-015-0155-1 (PMC4410453; doi:10.1186/s13073-015-0155-1)
Supplement: Additional file 1: — Supplementary notes and methods. 1) Comparison of ClinSeK targeted alignment with BWA aln. 2) Computing ClinSeK variant score. 3) Read pileup and calculation of allele support. 4) Duplicate insert marking. 5) Somatic mutation detection by ClinSeK. 6) Variant calling on >1,000 normal samples. 7) Implementation and memory footprint. 8) Run time on targeted exome sequence samples. [file 13073_2015_155_MOESM1_ESM.docx]

# Comparison of ClinSeK targeted alignment with BWA

We compared the ClinSeK read alignment on the targets with the alignment produced by BWA aln (Li & Durbin, 2009). We focused on 1000 ClinVar heterozygous sites randomly chosen from 700 samples and forced ClinSeK to consider these sites as variant sites and compared the alignment with those produced by BWA aln. We considered only reads that were assigned with high mapping quality (>30) by either BWA or ClinSeK and high base quality (>30) at the target site. ClinSeK and BWA, respectively, aligned 1141882 and 1137621 reads covering the selected target site. There were 1137012 reads in common between the two alignments as determined by read name and whether the read was the first in the pair. The number of reads aligned by only ClinSeK was 4870; the number of reads aligned by only BWA was 609. The concordance rate between the alignments produced by the two programs was 99.52% (Figure 2A). We used BLAT^1^ (35x1 [2009/02/26], default parameter) to cross-validate the alignment produced solely by either program. Of the 4870 read alignments produced by ClinSeK, 4817 alignments can be validated as top hits from BLAT results. Only 13 reads were mapped by BLAT to other regions of the genome with higher alignment scores. The rest of the reads were of low quality but were rescued through mate alignment. BWA-specific mapping was validated by BLAT for 606 of the 609 read alignments produced by BWA (See Figure S9 for the distribution of the BLAT scores of the BWA-specific reads). We also observed that 306 out of the 609 reads were positioned on the boundary of the aligned region by BLAT (<10bp to the end of the reads). In fact, in 125 reads, the target site was out of the aligned region from BLAT. This indicates that about half of the ClinSeK false negatives were due to potential extension by BWA of sequences that were mal-aligned to the target site. Use BLAT as reference does not provide an absolute validation because the origins of those reads are essentially unknown. However, having consistent alignment with a widely used independent aligner does indicate that ClinSeK achieved likely better alignment quality than BWA align.

**Figure S9** **Comparison between ClinSeK alignment with BWA aln.** Shown is a histogram of the alignment scores for the reads aligned by only BWA but not by ClinSeK. The alignment scores were calculated by BLAT, with the maximum score of 200 for reads as long as 100 base pairs. Reads not covering the target sites with high base qualities were excluded from the plot.

In studying the discrepant mapping between ClinSeK and BWA, the following four observations can be made. 1) Most ClinSeK false negatives contain more than 5 mismatches that are evenly distributed through the reads. This is due to the ClinSeK’s seeding strategy. Care must be taken for using such low quality reads for variant calling in clinics. Some reads were absent because only the low quality boundaries reach the target sites. 2) ClinSeK successfully maps more reads with multiple ‘N’ but high quality bases in the remaining sequence (see Figure 2B for the distribution of the alignment score of the reads missed by BWA). 3) The paralogous scanning strategy used by ClinSeK is effective in achieving highly specific read alignment at regions that contain paralogy in the reference. 4) ClinSeK tends to soft-clip reads when there are too many mismatches at the ends of the reads. This leads to more conservative variant calling at the target site.

# Computing ClinSeK variant score

In ClinSeK variant calling, two models are contrasted in computing the variant score: 1) reference model $M_{r}$: all variants are explained by sequencing error or contamination; and 2) variant model $M_{v}$: variants are explained jointly by sequencing error and the presence of a variant allele at fraction $f$. The p-value of calling a variant is computed by:

$$P\left( M_{r} | D \right)= \frac{P\left( D | M_{r} \right)P\left( M_{r} \right)}{P\left( D | M_{r} \right)P\left( M_{r} \right)+P\left( D | M_{v} \right)P\left( M_{v} \right)}$$

$$= \frac{\int_{0}^{c_{max}} P\left( D | M_{r}, c \right)P(M_{r})dP(c|M_{r})}{\int_{0}^{c_{max}} P\left( D | M_{r}, c \right)P(M_{r})dP(c|M_{r})+ \int_{0}^{1} P\left( D | M_{v},f \right)P\left( M_{v} \right)dP(f|M_{v})}$$

Here, $c$ denotes the extent of contamination. Without prior knowledge of the distribution of the allele frequency and contamination, we assume a uniform density in both cases, i.e., $P\left( f | M_{r} \right)=1$ and $P\left( c | M_{r} \right)=1/c_{max}$. The prior probability $P\left( M_{v} \right)=1-P\left( M_{r} \right)=\mu$ is the empirical mutation rate. The likelihood functions ($P\left( D | M_{r},c \right)$ and $P\left( D | M_{v},f \right)$) are given by a binomial distribution $Binom(n,p_{v})$ where $p_{v}$ is the expected number of reads that support the variant and $n$ is the total number of reads. Letting $e$ denote the sequencing error rate, $p$ for $P\left( D | M_{r},f \right)$ and $P\left( D | M_{v},f \right)$ has the following form,

$$p_{v}(c)={1-p}_{r}=c\left( 1-e \right)+(1-c)e$$

$$p_{v}\left( f \right)=1-p_{r}=f\left( 1-e \right)+\left( 1-f \right)e.$$

Here, we assume that the site is bi-allelic. Under such formulation, the likelihood takes the following form,

$$P\left( D | M_{r} \right)=\int_{0}^{c_{max}} P\left( D | M_{r}, c \right)dP\left( c | M_{r} \right)$$

$$=\binom{k_{v}+k_{r}}{k_{v}}\int_{0}^{c_{max}} {p_{v}}^{k_{v}}\left( 1-p_{v} \right)^{k_{r}}dc$$

$$=\binom{k_{v}+k_{r}}{k_{v}}\frac{1}{1-2e}\int_{p_{v}(0)}^{p_{v}(c_{max})} p^{k_{v}}\left( 1-p \right)^{k_{r}}dp$$

$$=\binom{k_{v}+k_{r}}{k_{v}}\frac{1}{1-2e}[B\left( p_{v}(c_{max}); k_{v}+1,k_{r}+1 \right)- B\left( p_{v}(0); k_{v}+1,k_{r}+1 \right)]$$

where $B(x;a,b)$ is the incomplete beta function.

$$P\left( M_{r} | D \right)=\frac{U}{U+V}$$

where

$$U=\left( 1-\mu\right)\left[ B\left( p_{v}\left( c_{max} \right); k_{v}+1,k_{r}+1 \right)- B\left( p_{v}\left( 0 \right); k_{v}+1,k_{r}+1 \right) \right]$$

$$V=\mu[B\left( p_{v}(1); k_{v}+1,k_{r}+1 \right)- B\left( p_{v}(0); k_{v}+1,k_{r}+1 \right)] c_{max}$$

A special case arises when $c_{max}=0$ or there is no contamination; the expression of the reference explanation has no marginalization:

$$P\left( M_{r} | D \right)= \frac{P\left( D | M_{r} \right)P\left( M_{r} \right)}{P\left( D | M_{r} \right)P\left( M_{r} \right)+ \int_{0}^{1} P\left( D | M_{v},f \right)P\left( M_{v} \right)dP\left( f | M_{v} \right)}$$

$$=\frac{U}{U+V}$$

where

$$U=\left( 1-\mu\right){p_{v}\left( 0 \right)}^{k_{v}}\left( 1-p_{v}\left( 0 \right) \right)^{k_{r}}$$

$$V=\mu[B\left( p_{v}(1); k_{v}+1,k_{r}+1 \right)- B\left( p_{v}(0); k_{v}+1,k_{r}+1 \right)]$$

# Read pileup and calculation of allele support

All the reads mapped to the target site are scanned for alleles present at the target site. We consider four types of alleles: 1) balanced substitutions for which the reference sequence and alternative sequence must have the same length; 2) insertion; 3) deletion; and 4) unbalanced substitutions for which the reference sequence and alternative sequence have different lengths. This can be viewed as a simple substitution contiguous to an insert or a deletion. We count the number of inserts that provide evidence for each allele found, excluding inserts that either support the allele but have low base quality, or support the allele at the end of the read. Different from GATK, MuTect and VarScan2, we report variants if there are nearby mutations or if there are more than two alleles at the site investigated, as long as we have high confidence in the alignment of the variant reads. This approach makes more sense if the user possesses prior knowledge that potential mutations on the site may be associated with human disease. This improves the sensitivity of ClinSeK to true mutations (Figure 3A).

In cancer clinics, accurate allele frequency is an important indicator of the heterogeneous structure of a tumor genome^2^. For paired-end sequencing, the entire inserts rather than the constituent reads are more natural units to use in calculating allele frequencies. This is in contrast to the approaches taken by, for example, the variant calling of MuTect^3^ and SAMtools,^4^ which report the number of reads rather than inserts. If the two mate-reads overlap and support the same allele, the support is counted only once. If the mate-reads do not agree, the disagreement is first resolved by comparing base qualities, and the resolved allele is considered in the pile-up outcome. Such practice further reduces the sequencing error by cross-validating two mate-reads that originate from the same insert.

# Duplicate insert marking

In clinical applications, sensitivity to variants, particularly low frequency mutations, is crucial to the detection of under-represented clones. The current practice of marking duplicate inserts (e.g., as implemented in SAMtools^4^ and Picard^5^), though fully justified by the amplification origin of the insert duplication, is an agnostic approach in relation to sequence identity. An arbitrary insert is chosen as a non-duplicate and all remaining inserts are marked as duplicates. In the deep sequencing experiments that are often conducted in cancer research, inserts that carry true variants may be marked as duplicates (sampling-induced duplication^6^) in an indeterminate manner (see below). To address this ambiguity, we developed a new duplicate insert-marking procedure that is aware of the subtlety in the sequence identity and base quality. This contrasts with the current practices of checking only the aligned coordinates and CIGAR string. The duplicate marking procedure starts by first identifying inserts for which constituent reads are mapped to the same genomic positions with the same CIGAR string. As shown in Figure S7, these inserts are then sorted by the sum of the base qualities. The inserts with the best base quality are regarded as non-duplicates. The remaining inserts are successively compared against all the non-duplicate inserts with higher sums of base qualities. The insert is marked as a duplicate if every high-quality base of the insert agrees with the corresponding base from at least one non-duplicate insert with higher sums of base qualities. Similar to existing approaches, ClinSeK favors high-quality base reads while choosing the non-duplicate insert from the set of identical inserts. However, in contrast to existing methods, at least one insert suggesting a high-quality difference is kept as a non-duplicate. For deep targeted sequencing, such sequence-aware duplication marking is more conservative and better at preserving the sequence diversity.


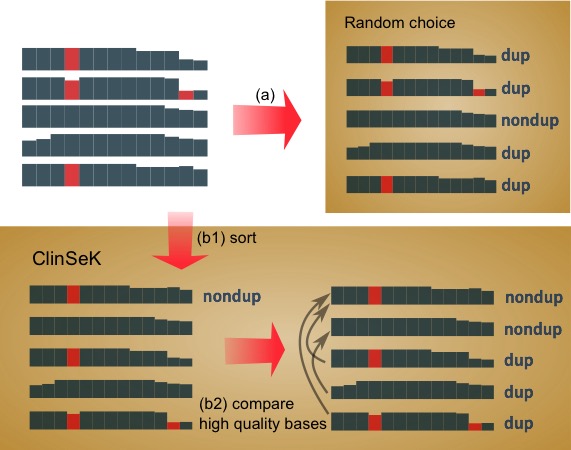


**Figure S7 Schematic diagram for marking duplicate inserts** Only one of the mate reads is shown for simplicity. All the reads shown are aligned to the same genomic coordinate with the same CIGAR string. (**a**) All reads are marked as duplicates except for a randomly chosen read. (**b**) The reads are first sorted by base qualities. Then a pairwise comparison between reads is performed such that read diversity at bases of high qualities is preserved.

To demonstrate the efficacy of duplicate marking with awareness of sequence identity, we randomly surveyed 1000 germline variants from 700 samples and plotted the fraction of unique inserts (in the sense of the same position, mate position and CIGAR string, but not sequence identity), which suggests different alleles at the target site if different members with the same configuration are marked as duplicates. As shown in Figure S8A, the proportion of affected inserts may range from 0% to 7%, which means that 0%–7% of the insert duplications may be due to sampling rather than PCR-amplification. For somatic mutations with low frequency (<10%), this has the potential to cause important mutations to be missed or the allele frequency to be underestimated. ClinSeK preserves the target base diversity of all these inserts in a deterministic way while effectively marking true PCR amplification duplicates (see Figure S8B for an example).


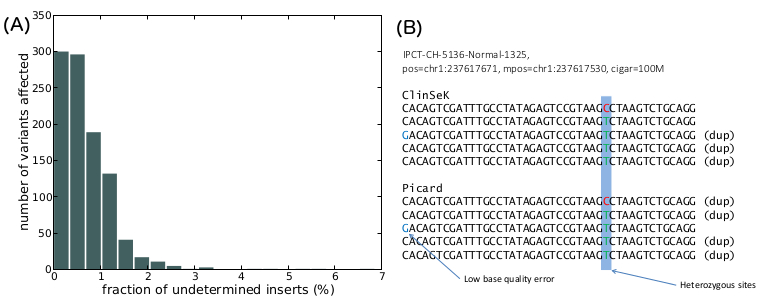


**Figure S8 Inserts duplicate in genomic coordinates but not suggesting different alleles** (A) Distribution of variants on which a certain extent of sampling-induced insert duplication causes undetermined variant calling from all inserts that share the same alignment. This illustrates the potential for distortion in variant allele frequency if duplicate inserts that share the same alignments are randomly marked without accounting for the sequence identity. (B) An example where random duplicate marking can lose the signal from bona fide genetic variants. The reads are all mapped to site chr1:237617671 and the mates to chr1:237617530 with CIGAR 100M. There is a heterozygous mutation (T) at the shaded column. Duplicate marking by randomly choosing the non-duplicate representative missed the signal of the mutation while choosing a suboptimal read (in terms of quality) with a sequencing error on another column.

# Somatic mutation detection by ClinSeK

**We compared the performance of ClinSeK in detecting somatic mutations on 719 clinically actionable sites from a dataset of 1049 targeted sequencing samples representing matched tumor and normal tissue samples. Compared with VarScan2 and MuTect, ClinSeK reported more somatic mutations. There were 35 AmpliSeq64 validated mutations that were reported by ClinSeK and VarScan but not by MuTect, and 13 were reported by ClinSeK and MuTect but not by VarScan2. This indicates that, for this dataset, ClinSeK has better sensitivity in reporting somatic mutations than the other two tools. The missed somatic mutations were largely of low allele frequency.**

# Variant calling on >1000 normal samples

We first studied germline mutations in 1060 samples on the 719 sites on the AmpliSeq64 panel. Altogether, ClinSeK identified 2702 variants: 1511 were identified as heterozygous variants, 485 were identified as homozygous variants, and the rest were identified as variants due to sample contamination. We also ran GATK haplotype caller on the same dataset. We found 2002 mutations, with 1519 heterozygous variants and 485 homozygous variants. As shown in Table S5, the confusion matrix indicates a high concordance rate (99.6%) between the results from ClinSeK and those from GATK haplotype caller. Of the 8 heterozygous variants identified by GATK, 5 had allele frequency lower than 15% and 3 had allele frequency higher than 88% (Table S6). This suggests that potential sample impurity may be responsible for these distorted allele frequencies (which deviate from 0.5 and 1). All 5 of the low frequency mutations were also found in the ClinSeK report, though they were genotyped to a homozygous reference with sample impurity. Of all the mutations reported, 3 mutations were indels. Both ClinSeK and GATK detected the 3 indels. We also compared the sites against 298 germline mutations experimentally assayed by AmpliSeq64. ClinSeK found all 298 mutations, reaching 100% sensitivity.

**Table S5** Confusion matrix of SNVs and indels identified by ClinSeK and the GATK haplotype caller on 719 AmpliSeq64 sites. 0/0: homozygous reference; 0/1: heterozygous variant; 1/1: homozygous variant.

|  | GATK Haplotype Caller | | |
| --- | --- | --- | --- |
| ClinSeK | 0/0 | 0/1 | 1/1 |
| 0/0 | NA | 5 | 0 |
| 0/1 | 0 | 1511 | 0 |
| 1/1 | 0 | 3 | 485 |

**Table S6** 8 Discordant genotypes between ClinSeK and GATK haplotype caller on 719 AmpliSeq64 sites: homozygous reference; 0/1: heterozygous variant; 1/1: homozygous variant.

| sample name | position | alt count | ref count | AF | ref | alt | ClinSeK genotype | GATK genotype |
| --- | --- | --- | --- | --- | --- | --- | --- | --- |
| IPCT_SQNM_01_1500-Normal-225-LI-Test | chr3:178927410 | 897 | 989 | 0.91 | A | G | 1/1 | 0/1 |
| IPCT-CH-1661-Normal-582 | chr5:112175770 | 551 | 627 | 0.88 | G | A | 1/1 | 0/1 |
| IPCT-CH-3606-Normal-1106 | chr4:55152040 | 610 | 690 | 0.88 | C | T | 1/1 | 0/1 |
| IPCT-CH-2325-Normal-556 | chr4:55152040 | 87 | 689 | 0.1263 | C | T | 0/0 | 0/1 |
| IPCT-CH-3606-Normal-1106 | chr7:55259435 | 65 | 622 | 0.1045 | T | C | 0/0 | 0/1 |
| IPCT_SQNM_01_1750-Normal-315 | chr5:149453051 | 29 | 262 | 0.1107 | C | T | 0/0 | 0/1 |
| IPCT-CH-1961-Normal-419 | chr17:7577539 | 90 | 728 | 0.1236 | G | A | 0/0 | 0/1 |
| IPCT-CH-1661-Normal-582 | chr4:55946242 | 81 | 1015 | 0.08 | C | T | 0/0 | 0/1 |

To further assess the performance of ClinSeK in detecting germline mutations, we also studied 9807 sites compiled from the ClinVar database and restricted to the 202 genes on the targeted sequencing panel. From 1375 samples, ClinSeK detected 98944 germline mutations (with 62242 genotyped as heterozygous variants and 36702 as homozygous variants) and GATK haplotype caller detected 98993 mutations (with 62428 genotyped as heterozygous variants and 36555 as homozygous variants). The confusion matrix also indicated high concordance between the two call-sets (99.7%) (Table S7). Similar to the results obtained for the AmpliSeq64 sites, discordances were due to potential sample impurity that results in variant allele frequencies that are distorted from the ideal levels of 0.5 and 1 (data not shown). All but 7 mutations genotyped by GATK as heterozygous variants were identified by ClinSeK as variants. Four of these 7 mutations contained long deletions (size >30 bp). The other 3 mutations were in regions of low coverage and were given a low quality score due to a lack of variant inserts. The differences between the results obtained from GATK and ClinSeK are due to the different ways the two programs count variants---GATK counts reads while ClinSeK counts inserts. This particularly matters when analyzing sites of low coverage. For example, if there is one variant insert but two variant mate-reads with overlapping regions that cover the target site, ClinSeK tends to conclude that the site is not a variant. A separate analysis focusing on indels reveals that ClinSeK is also highly sensitive to insertions and deletions. With the exception of the 4 long deletions mentioned above, ClinSeK reported all 14 insertions and 41 deletions identified by GATK. **The allele frequency reported by ClinSeK was also highly consistent with the values reported by GATK and VarScan2 (Figure S5A, B).**

**Table S7** Confusion matrix of SNV and indels identified by ClinSeK and GATK in calling germline mutations from 9807 ClinVar sites. 0/0: homozygous reference; 0/1: heterozygous variant; 1/1: homozygous variant.

| 1375 samples | GATK haplotype caller | | |
| --- | --- | --- | --- |
| ClinSeK | 0/0 | 0/1 | 1/1 |
| 0/0 | NA | 96 | 0 |
| 0/1 | 47 | 62192 | 3 |
| 1/1 | 0 | 150 | 36552 |

# Implementation and memory footprint

The software of ClinSeK is implemented in C and bundled with SAMtools API and a SIMD optimized Smith-Waterman library^7^ for local alignment. The memory requirement of ClinSeK is dependent on the number of target sites. ClinSeK caps the memory usage by storing the alignment and dumping a temporary intermediate file to disk. Therefore, the memory usage is independent of the number of reads. Processing 9807 ClinVar sites consumes less than 300MB of peak memory. The storage of the reference sequence around the target site and the k-mer hash table is responsible for most of the memory usage.

# Run time on targeted exome sequence samples

The run time scales almost linearly with the number of reads (Figure S9A). Processing a sample with 50 million reads takes around 20 minutes. The processing includes read alignment, duplicate marking, realignment and variant calling. We compared the run time of ClinSeK on the two target site lists we studied in this paper---719 AmpliSeq64 sites and 9807 ClinVar sites restricted to 202 cancer genes. Running ClinSeK when targeting the ClinVar sites took about twice the time needed to run ClinSeK on the same sample but when targeting the AmpliSeq64 sites (Figure S9B).


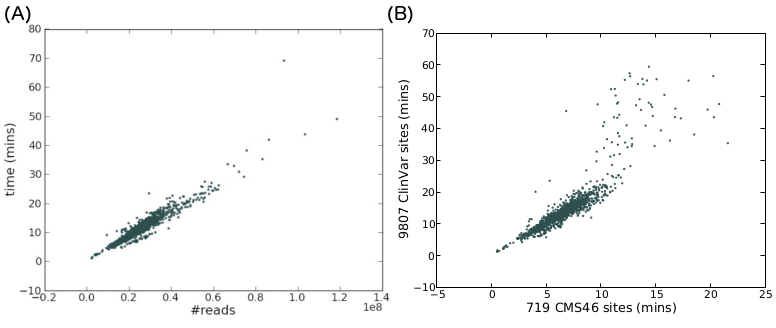


Figure S9 ClinSeK run time (A) ClinSeK run time vs sample size. Scatter plot of run time of ClinSeK vs sample size. Each dot corresponds to one of the 1225 deep sequencing samples. Sample size characterized by the number of containing reads is shown on the x-axis. (B) ClinSeK run time vs target sites size. Comparison as made in run time between ClinSeK targeting 719 AmpliSeq64 sites (x-axis) and 9807 ClinVar sites restricted to 202 cancer genes (y-axis).

GATK has an option that allows performing variant calling in only targeted regions and can achieve faster analysis. We thereby compared ClinSeK with this GATK targeted variant calling mode using 12.6 million reads sequenced from a cell line sample ([SRA accession: SRP033243]). We created a BED file, containing 100 bp intervals, each centering at one of the ClinVar sites targeted by ClinSeK. ClinSeK’s variant calling step alone was over twice as fast as GATK’s UnifiedGenotyper (8.7 seconds as compared to 23.8 seconds) with the BED file. When the entire processes (alignment and variant calling) were compared, ClinSeK was 77 times faster (129 seconds vs. 10,031 seconds) than BWA followed by GATK UnifiedGenotyper with the BED file and was 113 fold faster (129 seconds vs 14,711 seconds) than BWA followed by GATK UnifiedGenotyper without the BED file.

1. Kent, W.J. BLAT--the BLAST-like alignment tool. *Genome Res* **12**, 656-64 (2002).

2. Carter, S.L. *et al.* Absolute quantification of somatic DNA alterations in human cancer. *Nat Biotechnol* **30**, 413-21 (2012).

3. Cibulskis, K. *et al.* Sensitive detection of somatic point mutations in impure and heterogeneous cancer samples. *Nat Biotechnol* **31**, 213-9 (2013).

4. Li, H. *et al.* The Sequence Alignment/Map format and SAMtools. *Bioinformatics* **25**, 2078-9 (2009).

5. Harrow, J. *et al.* GENCODE: The reference human genome annotation for The ENCODE Project. *Genome Research* **22**, 1760-1774 (2012).

6. Zhou, W. *et al.* Bias from removing read duplication in ultra-deep sequencing experiments. *Bioinformatics* (2014).

7. Zhao, M., Lee, W.P., Garrison, E.P. & Marth, G.T. SSW library: an SIMD Smith-Waterman C/C++ library for use in genomic applications. *PLoS One* **8**, e82138 (2013).
