# Supplementary figures and images for: ClinSeK: a targeted variant characterization framework for clinical sequencing
Source: Genome Med. 2015 Mar 31;7(1):34. doi: 10.1186/s13073-015-0155-1 (PMC4410453; doi:10.1186/s13073-015-0155-1)

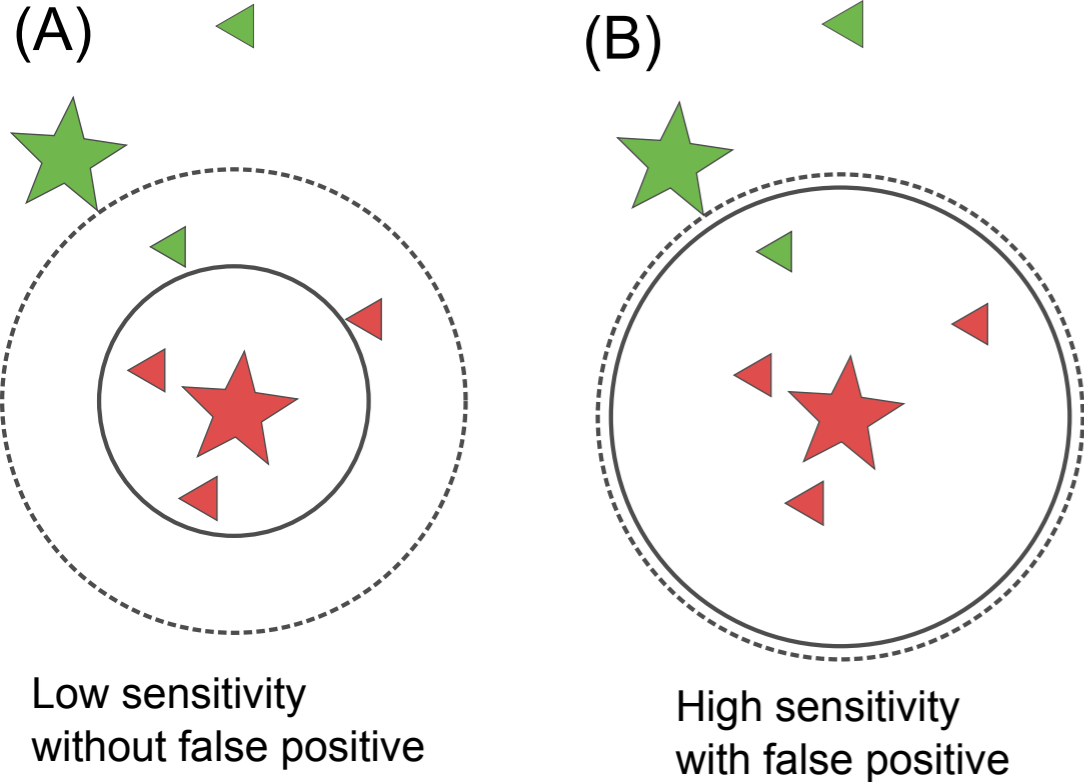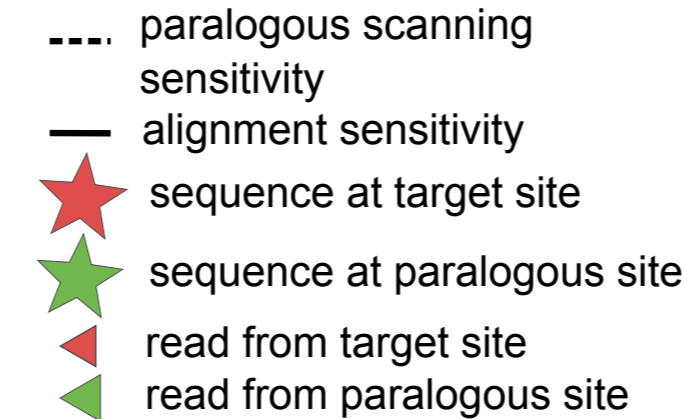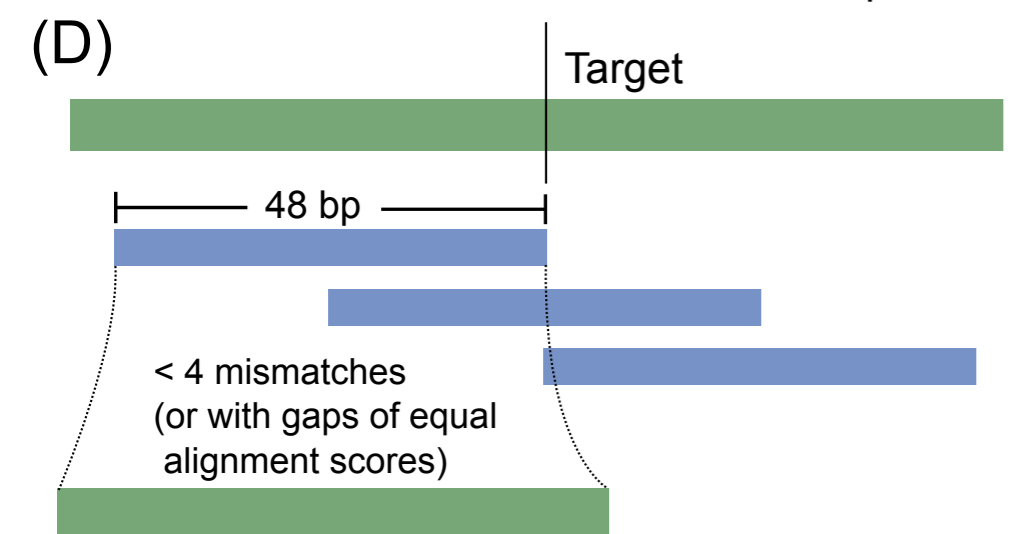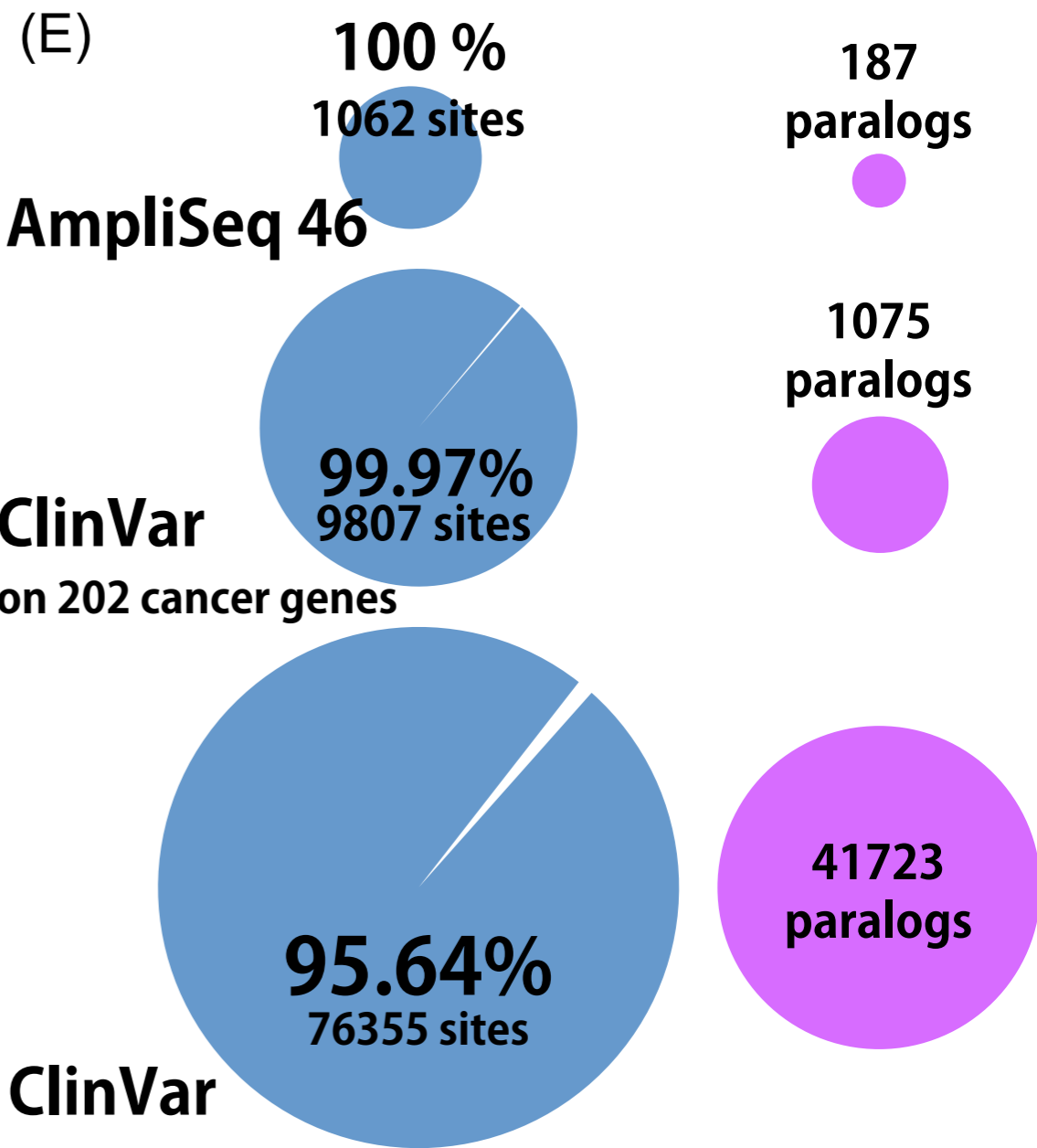

Supplement: Additional file 2: Figure S1. — Schematic illustration of paralogous scanning. Distance between symbols (triangles and stars) reflects the edit distance between sequences. Red star, target site sequence; red triangle, read sequenced from target site; green star, paralogous site sequence; green triangle, read sequenced from paralogous site. Size of the dashed circle indicates the sensitivity in identifying sites paralogous to the target site. Size of the solid circle indicates the sensitivity in read mapping. Greater circle size represents higher sensitivity. (A) Low sensitivity in read mapping and low sensitivity in paralogous scanning. No false positive exists (no green triangle in the solid circle), but there is a missing read alignment (red triangle outside the solid circle). (B) High sensitivity in read mapping and low sensitivity in paralogous scanning. False positives occur (green triangle in a solid circle). (C) High sensitivity in read mapping and high sensitivity in paralogous scanning. Neither false positives nor false negatives occur. (D) Definition of paralogous sites. Blue horizontal bar: sequence stretch on which fewer than four mismatches exist. Green horizontal bar: target sequence (top) and paralogous site sequence (bottom). We use a default scoring system with affine gap penalties of 2:3:1 for [mismatch]:[gap opening]:[gap extension]. (E) The number of target sites amenable to ClinSeK processing is shown in blue and the number of paralogous sites identified is shown in pink. Three different target site sets are studied: 1) AmpliSeq64; 2) ClinVar sites restricted to 202 cancer genes; 3) ClinVar sites. [file 13073_2015_155_MOESM2_ESM.pdf]

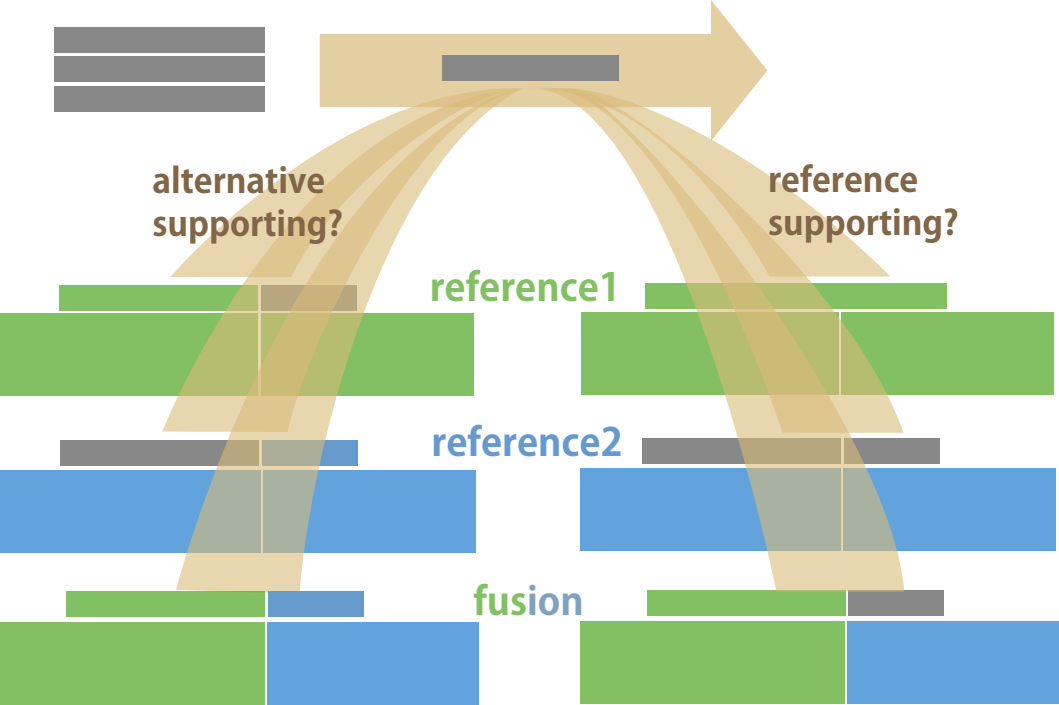

Supplement: Additional file 3: Figure S2. — Schematic diagram of fusion detection. The number of reads aligned to alternative fusion breakpoint assembly is contrasted with the number of reads aligned to the reference sequence around the breakpoint. Green and blue indicate the two reference sequences involved in the gene fusion. Narrower bars stand for short reads. The color of the reads indicates sequence similarity with the reference sequence. [file 13073_2015_155_MOESM3_ESM.pdf]

# IPCT-CH-4522-Tumor-1082

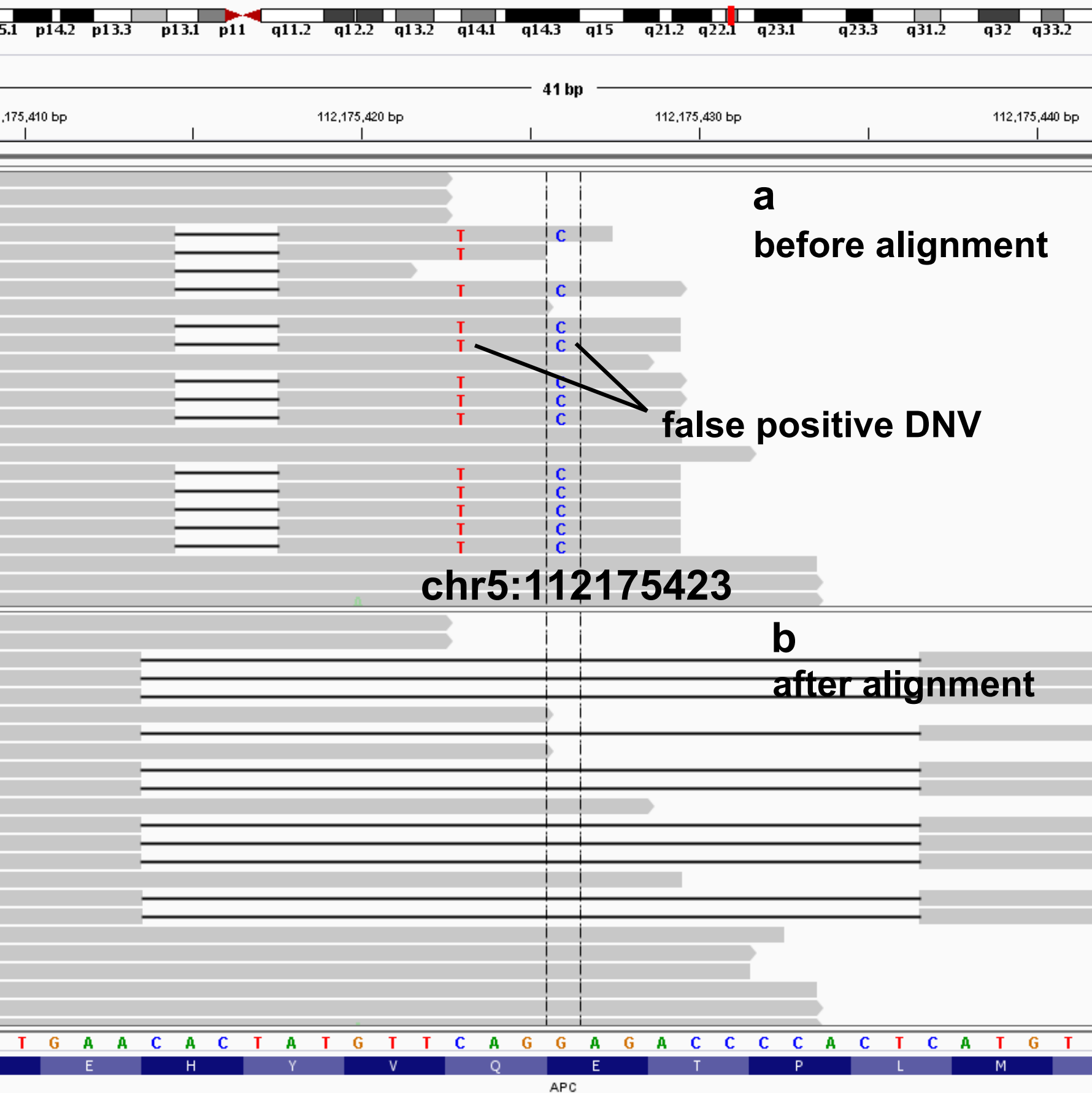

Supplement: Additional file 5: Figure S4. — Elimination of false positive SNVs by indel realignment Sample: IPCT-CH-4522-Tumor-1082; site: chr5:112175423. (a) Before indel realignment, a false positive mutation (T) was present. (b) After indel realignment, the false positive is eliminated. [file 13073_2015_155_MOESM5_ESM.pdf]

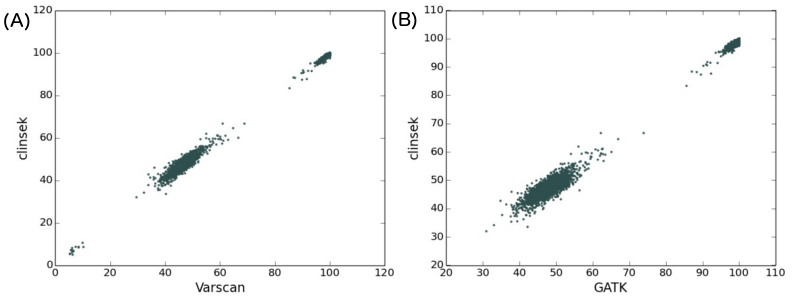

Supplement: Additional file 10: Figure S5. — Comparison of variant allele frequencies. (A) The variant allele frequencies are estimated by VarScan2 (x-axis) and ClinSeK (y-axis) from 3,472 genetic variants in 46 deep sequenced normal samples. (B) Variant allele frequencies estimated by GATK (x-axis) and ClinSeK (y-axis) from 3,467 germline mutations in 46 deep sequenced normal samples. [file 13073_2015_155_MOESM10_ESM.jpeg]
