# Supplementary material for: ClinSeK: a targeted variant characterization framework for clinical sequencing
Source: Genome Med. 2015 Mar 31;7(1):34. doi: 10.1186/s13073-015-0155-1 (PMC4410453; doi:10.1186/s13073-015-0155-1)
Supplement: Additional file 4: Figure S3. — Elimination of false positive SNVs by indel realignment. Sample: IPCT-CH-4335-Tumor-945; site: chr5:112175216. (a) Before indel realignment, a false positive mutation (T) was present. (b) After indel realignment, the false positive is eliminated. [file 13073_2015_155_MOESM4_ESM.pdf]

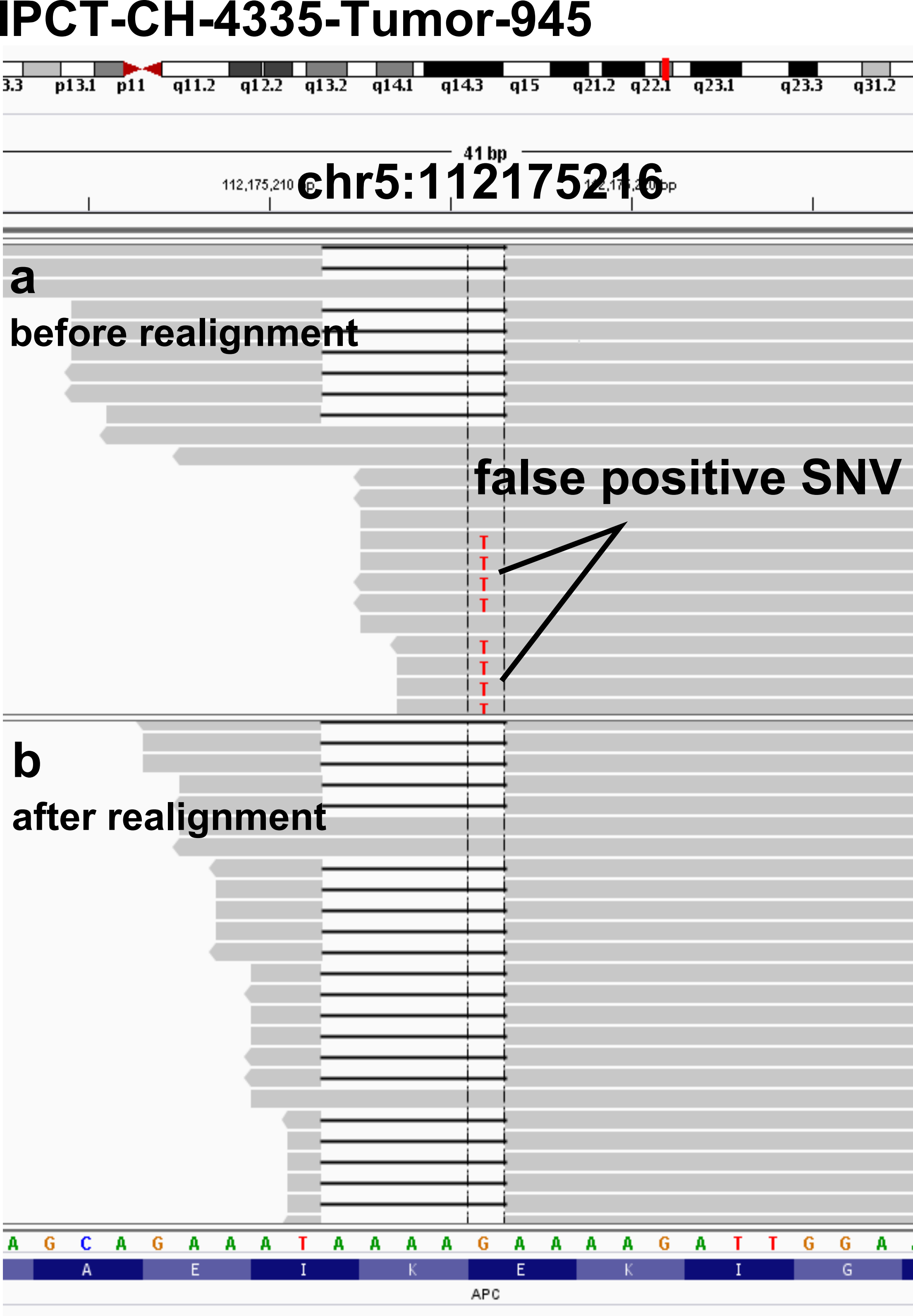
IPCT-CH-4335-Tumor-945
p13.1
p11
q11.2
q12.2
q13.2
q14.1
q14.3
q15
q21.2
q22.1
q23.1
q23.3
q31.2
41 bp
112,175,210 bp
chr5:112175216
a
before realignment
false positive SNV
b
after realignment
APC
