# Supplementary material for: ClinSeK: a targeted variant characterization framework for clinical sequencing
Source: Genome Med. 2015 Mar 31;7(1):34. doi: 10.1186/s13073-015-0155-1 (PMC4410453; doi:10.1186/s13073-015-0155-1)
Supplement: Additional file 6: Table S1. — List of validated mutations detected by only ClinSeK and missed by VarScan2 and MuTect, together with potential causes of missed mutations as reported from MuTect in rejecting these somatic mutations. [file 13073_2015_155_MOESM6_ESM.docx]

| sample | chrm | pos | gene | cpos | tumor alt | normal alt | reason reported by MuTect | VAF as reported by ClinSeK |
| --- | --- | --- | --- | --- | --- | --- | --- | --- |
| IPCT-CH-0174-Tumor-686 | chr12 | 25398284 | KRAS | 12 | 76 | 0 | nearby_gap_events | 29.34% |
| IPCT_SQNM_01_1705-Tumor-336 | chr17 | 7579359 | TP53 | 110 | 46 | 0 | nearby_gap_events | 12.24% |
| IPCT-CH-4364-Tumor-901 | chr7 | 140481417 | BRAF | 464 | 12 | 0 | possible_contamination | 2.081% |
| IPCT-CH-2088-Tumor-428 | chr17 | 7578550 | TP53 | 127 | 23 | 0 | strand_artifact | 43.75% |
| IPCT-CH-1840-Tumor-376 | chr4 | 153247289 | FBXW7 | 505 | 11 | 0 | possible_contamination | 2.534% |
| IPCT-CH-4747-Tumor-1067 | chr7 | 55241707 | EGFR | 719 | 68 | 0 | clustered_read_position | 8.269% |
| IPCT-CH-4747-Tumor-1067 | chr7 | 55241677 | EGFR | 709 | 52 | 0 | clustered_read_position | 8.448% |
| IPCT-CH-1997-Tumor-449 | chr4 | 55141036 | PDGFRA | 561 | 35 | 0 | clustered_read_position | 5.224% |
| IPCT-CH-3789-Tumor-1014 | chr17 | 7578389 | TP53 | 181 | 13 | 0 | possible_contamination | 2.234% |
