# Supplementary material for: ClinSeK: a targeted variant characterization framework for clinical sequencing
Source: Genome Med. 2015 Mar 31;7(1):34. doi: 10.1186/s13073-015-0155-1 (PMC4410453; doi:10.1186/s13073-015-0155-1)
Supplement: Additional file 7: Table S2. — List of validated mutations missed by MuTect but reported by ClinSeK and VarScan2. Potential causes of missed mutations are listed in column 6. It can be seen from these tables that MuTect misses high frequency somatic mutations because of a low-level alternative read count in the normal sample. [file 13073_2015_155_MOESM7_ESM.docx]

| sample | chrm | pos | tumor alt | normal alt | Reason reported by MuTect |
| --- | --- | --- | --- | --- | --- |
| IPCT-CH-3552-Tumor-752 | chr17 | 7577524 | 138 | 11 | alt_allele_in_normal |
| IPCT-CH-3462-Tumor-760 | chr17 | 7578406 | 211 | 2 | alt_allele_in_normal |
| IPCT-CH-4650-Tumor-1121 | chr7 | 140453136 | 163 | 2 | alt_allele_in_normal |
| IPCT-CH-0967-Tumor-499 | chr2 | 209113113 | 46 | 3 | alt_allele_in_normal |
| IPCT-CH-3059-Tumor-616 | chr17 | 7577538 | 219 | 2 | alt_allele_in_normal |
| IPCT-CH-3738-Tumor-1111 | chr12 | 25398284 | 51 | 2 | alt_allele_in_normal |
| IPCT-CH-2737-Tumor-799 | chr3 | 178952085 | 135 | 0 | nearby_gap_events |
| IPCT-CH-4524-Tumor-1128 | chr7 | 140453136 | 447 | 2 | alt_allele_in_normal |
| IPCT-CH-1336-Tumor-406 | chr17 | 7577094 | 75 | 1 | nearby_gap_events |
| IPCT-CH-4651-Tumor-1140 | chr17 | 7578534 | 387 | 2 | alt_allele_in_normal |
| IPCT-CH-3344-Tumor-885-A | chr7 | 140453137 | 332 | 2 | alt_allele_in_normal |
| IPCT-CH-4438-Tumor-968 | chr5 | 112175639 | 82 | 4 | alt_allele_in_normal |
| IPCT-CH-4524-Tumor-1128 | chr17 | 7578212 | 140 | 1 | triallelic_site |
| IPCT-CH-1591-Tumor-422-A | chr17 | 7577545 | 262 | 0 | nearby_gap_events |
| IPCT_SQNM_01_1442-Tumor-281 | chr12 | 25398284 | 153 | 3 | alt_allele_in_normal |
| IPCT-CH-4734-Tumor-1144 | chr17 | 7578263 | 275 | 2 | alt_allele_in_normal |
| IPCT_SQNM_01_1582-Tumor-285 | chr3 | 178936091 | 86 | 2 | alt_allele_in_normal |
| IPCT-CH-5025-Tumor-1224 | chr3 | 41266097 | 67 | 2 | alt_allele_in_normal |
| IPCT-CH-4547-Tumor-1035 | chr4 | 55953823 | 215 | 1 | nearby_gap_events |
| IPCT-CH-4540-Tumor-1087 | chr17 | 7578263 | 169 | 2 | alt_allele_in_normal |
| IPCT-CH-3721-Tumor-747 | chr17 | 7578553 | 288 | 2 | alt_allele_in_normal |
| IPCT-CH-4667-Tumor-1060-A | chr10 | 43617416 | 337 | 2 | alt_allele_in_normal |
| IPCT-CH-4545-Tumor-1131 | chr7 | 140453136 | 207 | 3 | alt_allele_in_normal |
| IPCT-CH-4831-Tumor-1044 | chr1 | 115256530 | 310 | 5 | alt_allele_in_normal |
| IPCT-CH-4328-Tumor-908 | chr17 | 7577551 | 155 | 2 | nearby_gap_events,alt_allele_in_normal |
| IPCT-CH-3530-Tumor-768 | chr10 | 89720702 | 201 | 1 | nearby_gap_events |
| IPCT-CH-3514-Tumor-766 | chr12 | 121431395 | 92 | 2 | alt_allele_in_normal |
| IPCT-CH-1084-Tumor-567 | chr12 | 25398284 | 18 | 0 | triallelic_site |
| IPCT-CH-4036-Tumor-1013 | chr3 | 178916854 | 193 | 0 | nearby_gap_events |
| IPCT-CH-4347-Tumor-920 | chr17 | 7577538 | 220 | 6 | alt_allele_in_normal |
| IPCT-CH-4755-Tumor-1134 | chr17 | 7578406 | 229 | 3 | alt_allele_in_normal |
| IPCT-CH-4420-Tumor-877 | chr17 | 7578406 | 555 | 2 | alt_allele_in_normal |
| IPCT-CH-4646-Tumor-1133 | chr17 | 7577120 | 155 | 2 | alt_allele_in_normal |
