# Supplementary material for: ClinSeK: a targeted variant characterization framework for clinical sequencing
Source: Genome Med. 2015 Mar 31;7(1):34. doi: 10.1186/s13073-015-0155-1 (PMC4410453; doi:10.1186/s13073-015-0155-1)
Supplement: Additional file 8: Table S3. — List of validated mutations missed by Varscan2 but reported by ClinSeK and MuTect. Potential causes of missed mutations obtained from manual inspection are listed in column 5. VarScan’s false negatives are primarily due to either 1) mutations that are found in regions where there are other mutations nearby (where mutations are locally clustered); or 2) the allele frequency being below a certain cutoff. Abbreviations: DNV, di-nucleotide variation; SNV, single nucleotide variation; TNV, tri-nucleotide variation. [file 13073_2015_155_MOESM8_ESM.docx]

| sample | chrm | pos | allele frequency | possible reason of missing |
| --- | --- | --- | --- | --- |
| IPCT-CH-0174-Tumor-686 | chr12 | 25398281 | 0.280303 | DNV |
| IPCT-CH-3951-Tumor-793-B | chr18 | 48604707 | 0.039593 | below AF cutoff |
| IPCT-CH-4329-Tumor-967 | chr3 | 178936091 | 0.027125 | close to another snv |
| IPCT-CH-1698-Tumor-370 | chr12 | 25398284 | 0.047319 | below AF cutoff |
| IPCT-CH-0314-Tumor-688 | chr12 | 25398284 | 0.051282 | below AF cutoff |
| IPCT-CH-4440-Tumor-889 | chr17 | 7578196 | 0.537794 | DNV |
| IPCT-CH-2691-Tumor-858 | chr5 | 112175184 | 0.067797 | TNV |
| IPCT-CH-3789-Tumor-1014 | chr18 | 48591919 | 0.027826 | below AF cutoff |
| IPCT-CH-3755-Tumor-878 | chr2 | 209113112 | 0.044747 | below AF cutoff |
| IPCT-CH-0646-Tumor-697 | chr3 | 178952085 | 0.165714 | DNV |
| IPCT-CH-2691-Tumor-858 | chr5 | 112175235 | 0.052632 | TNV |
| IPCT_SQNM_01_1489-Tumor-282 | chr4 | 55599338 | 0.553325 | DNV |
| IPCT-CH-2680-Tumor-1057-B | chr7 | 55259434 | 0.04142 | below AF cutoff |
| IPCT-CH-3728-Tumor-978 | chr3 | 178936091 | 0.025704 | below AF cutoff |
| IPCT_SQNM_01_1489-Tumor-282 | chr4 | 55599351 | 0.553134 | DNV |
| IPCT-CH-4751-Tumor-1081 | chr17 | 7578530 | 0.041257 | below AF cutoff |
| IPCT-CH-1007-Tumor-666 | chr12 | 25398284 | 0.029801 | below AF cutoff |
| IPCT-CH-1007-Tumor-666 | chr18 | 48593417 | 0.03876 | below AF cutoff |
| IPCT-CH-2691-Tumor-858 | chr5 | 112175246 | 0.0553 | TNV |
